# CUL4A ubiquitin ligase: a promising drug target for cancer and other human diseases

**DOI:** 10.1098/rsob.130217

**Published:** 2014-02-12

**Authors:** Puneet Sharma, Alo Nag

**Affiliations:** Department of Biochemistry, University of Delhi, South Campus, New Delhi, India

**Keywords:** CUL4A, cancer, genomic stability, cell cycle

## Abstract

The ability of cullin 4A (CUL4A), a scaffold protein, to recruit a repertoire of substrate adaptors allows it to assemble into distinct E3 ligase complexes to mediate turnover of key regulatory proteins. In the past decade, a considerable wealth of information has been generated regarding its biology, regulation, assembly, molecular architecture and novel functions. Importantly, unravelling of its association with multiple tumours and modulation by viral proteins establishes it as one of the key proteins that may play an important role in cellular transformation. Considering the role of its substrate in regulating the cell cycle and maintenance of genomic stability, understanding the detailed aspects of these processes will have significant consequences for the treatment of cancer and related diseases. This review is an effort to provide a broad overview of this multifaceted ubiquitin ligase and addresses its critical role in regulation of important biological processes. More importantly, its tremendous potential to be exploited for therapeutic purposes has been discussed.

## 2. Introduction

Covalent attachment of ubiquitin to cellular proteins is one of the major post-translational modifications (PTMs) that play a vital role in regulating cellular physiology. This process, called ubiquitylation or ubiquitination, is mediated by a cascade of enzymatic reactions involving E1, E2 and E3 enzymes (see appendix A). The selectivity of ubiquitination resides in the specificity of E3 ligases for their substrate. Based on the structure of the catalytic core, two main classes of E3s identified are HECT (homologous to E6-AP C-terminus) and RING (really interesting new gene). A superfamily of RING-based E3 ligases consists of an evolutionarily conserved protein, called cullin, which acts as a scaffold and recruits a RING-based protein at one end to form a catalytic core and cullin-specific adaptor and/or substrate receptor at the other end. The whole complex, called cullin–RING ubiquitin ligase (CRL), owing to its modularity is able to switch its adaptor and/or substrate receptor, thereby targeting substrates involved in diverse cellular processes.

**Figure 1.**
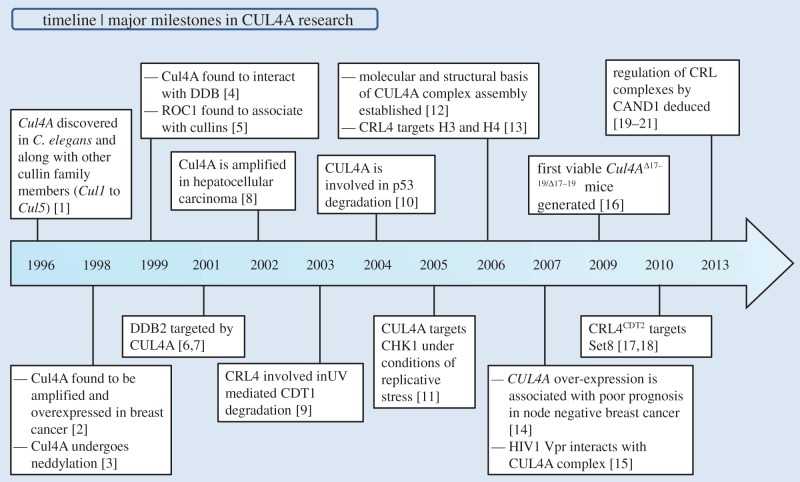
Timeline highlighting crucial discoveries that provided insights into CUL4A functions, regulation and association with various pathologies.

The human genome encodes six members of the cullin family (CUL 1, 2, 3, 4A, 4B and 5) that are characterized by a cullin homology domain present towards the C-terminal, and two atypical cullins (CUL7 and CUL9) that consist of additional homology domains. Among the six cullins, the CUL4 subfamily comprises two members, CUL4A and CUL4B, which share 83% sequence identity and functional redundancy. CUL4A was discovered along with CRL1 E3 ligases, better known as the SCF (S-phase kinase-associated protein 1 (SKP1)–cullin 1 (CUL1)–F-box protein) complex that serves as the archetype for the CRL family [1]. The initial observation of its overexpression in breast cancer accelerated the quest for finding its normal function in the cell [2]. Subsequent active research spanning over a decade has highlighted the role of CUL4A complexes in regulating substrates involved in the cell cycle, signalling, tumour suppression, DNA damage response and chromatin remodelling (figure 1). Even though both CUL4A and CUL4B share extensive homology and functional redundancy, it is CUL4A that has drawn much attention owing to its association with oncogenesis.

This review intends to summarize recent insights into functioning of the CUL4A complex and its regulation. We also emphasize the findings demonstrating CUL4A's association with oncogenesis and its importance as a prognostic marker and a predictor of drug response. We finally contend that CUL4A can serve as an attractive target for therapeutic intervention in various human diseases.

## 3. *Cul4A* phylogeny and organization

**Figure 2.**
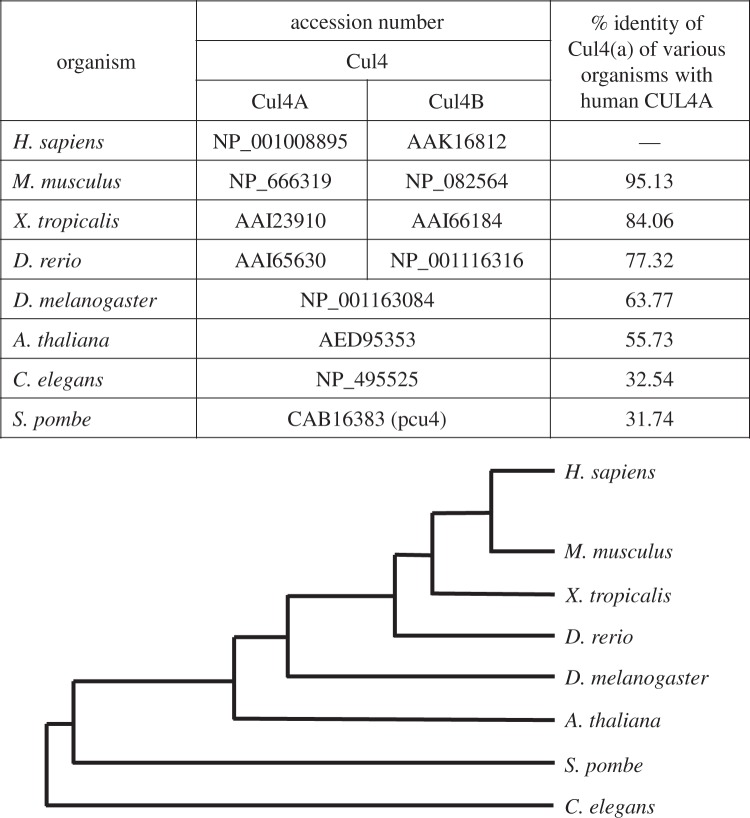
Phylogenetic analysis of CUL4A protein in eukaryotic species. The table compares sequence identity of Cul4A of various eukaryotes with human CUL4A. Below the table, the phylogenetic tree represents the evolutionary relationship between these organisms. Relationship was inferred using PHYLIP (Kitsch) program and the tree was visualized using PhyloDraw. Depicted here is a schematic representation.

CRL complexes are of ancient origin. Extensive phylogenetic analysis revealed the existence of three ancestral cullin genes, named *Culα*, *Culβ* and *Culγ*, from which the modern cullin genes evolved after the unikont/bikont split [22]. It was also shown that *Cul4a/4b* evolved from the *Culγ* gene [22]. Higher eukaryotes such as *Homo sapiens*, *Mus musculus*, *Xenopus tropicalis* and *Danio rerio* have been found to contain CUL4A and CUL4B, whereas no such duplication is observed in the case of *Caenorhabditis elegans*, *Drosophila melanogaster* and *Arabidopsis thaliana*, suggesting that this genetic redundancy might be unique to higher eukaryotes. This hypothesis may also be partially supported by the observation that human CUL4A shares high sequence identity with Cul4A of other higher eukaryotes (figure 2). In addition, all the known major functions of Cul4 have been found to be conserved from lower to higher eukaryotes. However, in higher eukaryotes Cul4A and Cul4B also perform specialized functions despite their high sequence identity. For example, CUL4B, but not CUL4A, has been shown to target oestrogen receptors and peroxiredoxin III [23,24]. Additionally, *Cul4B* plays an important role in embryonic development as *Cul4B*^Δ3–5/Δ3–5^ mice, having deletion of exons 3–5, exhibit embryonic lethality. Furthermore, *Cul4B* heterozygotes show severe developmental delay, which may be ascribed to disorganized placenta with damaged vascularization in these mutants [25]. However, no such obvious abnormalities are apparent in *Cul4A* null mice [16,26,27].

In humans, *CUL4A* is a single-copy gene consisting of 20 exons and is mapped at 13q34 chromosomal segment. It encodes four transcript variants that finally translate into three isoforms. Transcript-1 is the longest and dominant form and encodes isoform-1 of 759 amino acid residues and is the focus of the review. Transcripts 2- and -3 use an alternative 5′-terminal exon, compared with variant-1, resulting in isoform-2 of 659 amino acid residues with a shorter N-terminus. Transcript-4 also uses an alternative 5′ terminal exon, but along with an alternative in-frame splice junction, compared with variant-1. The isoform-3 encoded by this variant is 667 amino acids long and consists of a shorter N-terminus and an alternative internal segment compared with isoform-1.

## 4. Structural insights into CUL4A complex

CUL4A is an 87-kDa protein and exhibits elongated structure with an arc-shaped helical N-terminal domain that binds to a substrate receptor or substrate binding adaptor and a globular C-terminal domain that binds the small RING finger protein ROC1 (ring of cullins) [4,12]. ROC1 associates with the conserved C-terminal domain of CUL4A and helps in recruitment of E2 enzyme to the cullin complex. Although this catalytic core remains the same in CRLs, each cullin recruits its specific adaptor, e.g. F-box, BTB or SOC/BC-box. However, CUL4A uses a 127-kDa cellular protein, DNA damage binding protein 1 (DDB1), which can perform dual functions of adaptor or substrate binding receptor [5]. Structurally, DDB1 consists of 21 WD40-like repeats that fold into three β-propeller (BP) domains, namely BPA, BPB and BPC, and a helical C-terminal domain. Detailed crystallographic analysis of the DDB1–CUL4A–ROC1 apparatus revealed that DDB1 BPB interacts with CUL4A, while a BPA–BPC double propeller forms a clam-shaped binding pocket for substrate or substrate receptor that faces towards the E2-attachment site of ROC1. BPB association with CUL4A involves two separate interfaces. CUL4A uses the tip of its N-terminal domain and helices 2 and 5, respectively, to interact with those interfaces. Specifically, residues 82–85, 87, 88, 91, 92, 150–152, 154, 155, 158, 159 and 162 on DDB1 were found to be crucial for the DDB1–CUL4A interaction, and disruption of these residues leads to weaker complex formation [12,28].

The endogenous CUL4 substrate receptors having WD40 repeats, WDXR motifs or DDB boxes are referred to as DDB1 and Cul4A-associated factors (DCAFs) or DDB1-binding WD40 (DWD) proteins or CDW-proteins (CUL4 and DDB1 associated WDR proteins) [12,29–31]. These substrate receptors of CUL4A contain various protein–protein interaction domains which selectively interact with motifs called ‘degrons’ present on the substrate. It is by switching these diverse substrate receptors that CUL4A complex can recruit a repertoire of substrates for ubiquitination. However, functions of most of these DCAFs are yet to be explored.

DDB2 and Cockayne syndrome A (CSA) proteins are two well-known bifunctional DDB1-interacting proteins that act as substrate receptors for CUL4A and damage detection proteins in the nucleotide excision repair (NER) process. Being substrate receptors, DDB2 and CSA are likely to also play a role in the regulation of CUL4A function. Additionally, their complexes with DDB1 exhibit high similarity even though they share limited sequence identity. DDB2 tethers with DDB1 by inserting its N-terminal helix–loop–helix (HLH) motif between the DDB1 BPA–BPC double propeller and binds to DNA using its BP domain [32]. Similarly, CSA also uses the HLH motif to bind to DDB1 BPA–BPC double propeller and may use sides of BPs opposite to DDB1 to recognize substrates for ubiquitination [33].

Elucidation of CUL4A complex structure with DDB2 and CSA are just the initial strides in our understanding of structural logic behind some of its functions, knowledge of which is still incomplete. Thus, thorough analysis of CUL4A structural complexes may help in providing novel insights regarding its mechanism of action and its regulation.

## 5. CUL4A plays important role in maintaining cellular physiology

CUL4A complex has been known to target a multitude of regulatory proteins, thereby exerting its effect on important cellular processes. In general, it is involved in cell cycle regulation and maintenance of genomic stability. However, it may perform specialized functions in particular tissues, which is evident from its role in haematopoiesis and spermatogenesis. High expression of Cul4A has been found in testis and spleen, and also in heart and skeletal muscles, wherein Cul4B expression has been found to be considerably low, which further substantiates the fact that CUL4A might not have complete functional redundancy with CUL4B [34].

### 5.1. Regulation of cell cycle

The key cellular events of the mammalian cell cycle are precisely regulated by undulating activity of cyclins and their regulators. The oscillating activity of the cell cycle proteins is majorly regulated by the ubiquitin–proteasome system (UPS). CUL4A facilitates smooth S-phase progression by proteolysis of cyclin-dependent kinase (CDK) inhibitors (CDIs) and inhibiting re-replication of genomic DNA (discussed below). Among CDIs, p21^CIP1/WAF1^, p27^KIP1^ and p16^INK4a^ are regulated by CRL4 complex. *CUL4A* gene is cell cycle regulated, as genome-wide analysis of human fibroblast transcripts reveals its mRNA to be high at the G1/S boundary [35]. In addition, nuclear CUL4A levels show slight increase during G1 to S transition in synchronized HeLa cells [6]. Deletion of *Cul4A* in mouse embryonic fibroblasts (MEFs) leads to mild decrease in proliferation along with delay in S-phase entry, deficiency in M-phase progression, aberrant number of centrosomes, multipolar spindles and micronuclei formation, thereby corroborating its role in regulation of the cell cycle and genomic stability [36].

CRL4^CDT2^ mediates proteolysis of p21^CIP1/WAF1^, associated with chromatin bound proliferating cell nuclear antigen (PCNA) during S phase along with SCF complex which also degrades it at the G1/S boundary [37,38]. In unperturbed cycling cells, p21^CIP1/WAF1^ accumulates during G1 phase where it may promote cyclin-D/CDK4/6-dependent events and attenuates the activity of cyclin-E/CDK2 and cyclin-A/CDK2. In *Cul4A*^Δ17–19/Δ17–19^ knockout mice, increased stabilization of p21^CIP1/WAF1^ was observed, which enforced UV-responsive G1/S checkpoint, thereby helping the NER machinery to recognize moderate helix-distorting adducts [16].

CUL4A–DDB1 complex has also been reported to be involved in proteolysis of p27^KIP1^. Studies show that CUL4A–DDB1 complex can interact with either SKP2 or DDB2-Artemis to recruit p27^KIP1^ for ubiquitination and subsequent degradation [39–41]. However, *in vitro* ubiquitination of p27^KIP1^ still needs to be reported [39,40]. p27^KIP1^ has also been shown to be independently degraded by SCF^SKP2^ and KPC1/2 [42,43]. p27^KIP1^ inhibits the activity of cyclin-E/CDK2 during G_0_ and early G_1_ and plays a role in cell cycle exit. Interestingly, CUL4 complex in *Drosophila* has been shown to target cyclin E. However, in cell lines, only CUL4B was found to interact with endogenous cyclin E even though both CUL4A and CUL4B were able to polyubiquitinate cyclin E *in vitro* [44].

A recent study showing interaction of CUL4A with p16^INK4a^ promoter establishes another link with the cell cycle, because CDK inhibitor p16^INK4a^ is known for its functions in tumour suppression and cell ageing processes [45]. It was observed that CUL4A–DDB1 complex plays a crucial role in activation of p16^INK4a^ during oncogenic checkpoint response, and the effect is neutralized by polycomb repression complexes in normal cells. This might suggest a possible role of CUL4A in controlling p16^INK4a^ transcription.

Altogether, these pieces of evidence suggest that by controlling the degradation of key players, CUL4A helps in maintaining normal cell proliferation and survival under stressful conditions.

### 5.2. Maintenance of genomic stability

Genomic stability during cell cycle progression is maintained by controlling the fidelity of DNA replication, accurate distribution of chromosomes in daughter cells and efficient DNA repair and via check point controls. CUL4A plays a crucial role in this process by ensuring that the genome is replicated only once per cell cycle. Studies in *C. elegans* first demonstrated the involvement of CUL4 complex in preventing re-replication by degrading replication licensing factor CDT1 during S phase [46]. High levels of CDT1 as well as massive DNA re-replication were observed in proliferating cells containing inactivated CUL4 [46]. Later, CUL4 complex containing CDT2 as substrate recognition subunit in worms and humans was shown to target CDK inhibitor CKI-1 and p21^CIP1/WAF1^, respectively, as a part of the replication licensing mechanism [47].

**Figure 3.**
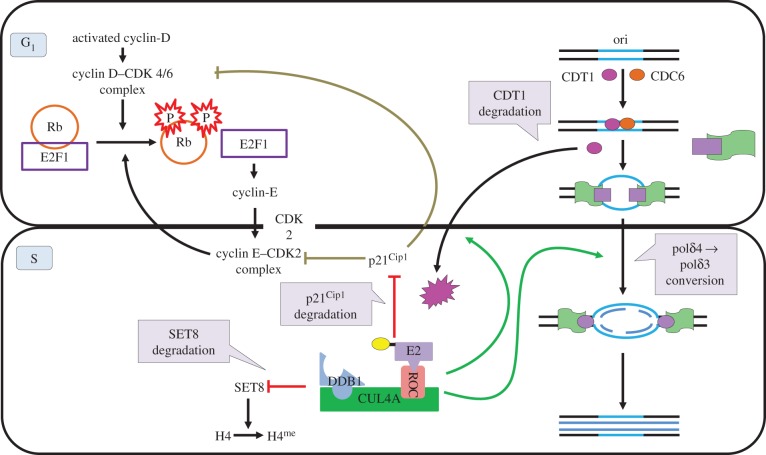
Role of CUL4A complex in progression of normal cell cycle. CUL4A complex ensures smooth progression of cell cycle by mediating degradation p21^CIP1/WAF1^, CDT1, SET8 and p12 subunit of polymerase δ. Degradation of p21^CIP1/WAF1^, which attenuates the activity of cyclin E-CDK2 enables S-phase entry. By targeting Set8 methyltransferase, CUL4A prevents ectopic chromosomal condensation during S phase. At genomic level, CUL4A complex targets CDT1 for degradation to prevent re-replication of the genome. In addition, degradation of the p12 subunit of DNA polymerase δ (pol δ4) converts it into active trimeric pol δ3 which may play a role in DNA replication and DNA repair.

During S phase, CDT1 binding to origin recognition complex acts as nucleation site for pre-replication complex formation. Once ori is licensed, CRL4^CDT2^ brings about the degradation of chromatin bound CDT1 to prevent further licensing [9,48,49]. Another factor that may contribute to re-replication is PR-Set7/Set8 histone H4K20 methyltransferase that accumulates during G_2_ and M phase. Monomethylation of lysine 4 of histone H4 (H4K20me^1^) carried out by Set8 methyltransferase promotes chromatin compaction, thereby allowing proper mitosis, and may hinder subsequent S-phase progression. CRL4^CDT2^ prevents premature accumulation of H4K20me1 at replication origins by degrading it during the S phase [17,18,50]. Furthermore, p12 subunit of heterotetrameric DNA polymerase δ (pol δ4) is degraded by CRL4^CDT2^ under normal as well as following UV irradiation to form trimeric pol δ3 which exhibits DNA repair properties (figure 3) [51].

CUL4A plays a vital role in maintaining genomic integrity by preventing replication of genomic DNA during genotoxic stress. Following DNA damage, CDT1 and PR-Set7/Set8 also undergo rapid proteolysis by CUL4A complex to prevent relicensing of ori and enhance transactivation function of p53. Additionally, p21^CIP1/WAF1^ also undergoes UV-induced degradation by CUL4A. p21^CIP1/WAF1^ is the key protein involved in mediating cell cycle arrest following DNA damage. It was observed that *Cul4A*^Δ17–19/Δ17–19^ MEFs exhibit accumulation of p21^CIP1/WAF1^ following UV irradiation leading to prolonged G_1_/S arrest, which may allow additional time for NER to rectify the damage [16]. Additionally, *Cul4A*^Δ17–19/Δ17–19^ mice were also found to be hyper-resistant to UV-B-induced skin carcinogenesis, and MEFs were unable to undergo G_2_ arrest, DNA re-replication and cell death [16]. These results highlight the physiological role of CUL4A in NER and tumourigenesis.

CRL4^DDB2^ and CRL4^CSA^ are two well-known CRL4 E3 ubiquitin ligases that participate in the evolutionarily conserved NER pathway. The NER pathway recognizes and corrects the helix-distorting DNA damage caused by cross-linking agents, mutagens and UV radiation. The bulky DNA adducts, generally cyclobutane–pyrimidine dimers (CPDs) and pyrimidine (6–4) pyrimidone photoproducts (6-4PPs), if not repaired by NER, hamper transcription and replication and lead to apoptosis [52,53]. Generally, the DNA helix experiences 7–9° kink or bend in the presence of CPDs which constitutes 70–80% of nucleosomal DNA damage, whereas 6-4PPs induce more prominent 44° bend, which comprises 20–30% of linker DNA damage [54–56]. The importance of NER is illustrated by the fact that mutations in genes coding for proteins involved in this pathway results in pathologies such as xeroderma pigmentosum (XP), Cockayne syndrome (CS) and trichothiodystrophy (TTD), which are characterized by UV sensitivity, neurological impairment, developmental complications and premature ageing and in the case of XP, increased risk of cutaneous neoplasm.

The eukaryotic NER system consists of two major pathways, global genome repair (GG-NER) and transcription-coupled repair (TC-NER), which differ in lesion recognition but converge to use a common set of proteins for the effector functions of lesion incision, oligonucleotide removal, gap regeneration and nick ligation. GG-NER interrogates the whole genome for helical distortions via lesion-sensing complexes, DDB1–DDB2 and XPC–hHR23B–CEN2 [57–59]. UV induces dissociation of CSN (constitutively photomorphogenic-9 (COP9) signalosome) from CUL4A and its translocation to chromatin, thereby activating CRL4 complex [60]. DDB2 scans the genome for bulky adducts via its conserved tripeptide Phe–Gln–His (FQH) hairpin present at one end of its BP opposite the DDB1-binding site. A lesion is recognized when the hairpin inserts into the minor groove of DNA leading to flipping out of damaged pyrimidine bases which are stabilized by a hydrophobic pocket at the DDB2 surface [32]. While lesions containing 6-4PPs are easily reached by repair machinery, accessing CPDs requires relaxation of the nucleosome. CRL4^DDB2^ ensures this by ubiquitination of histones (H2A, H3 and H4) at the sites of UV lesions [13,61]. Concomitantly, CRL4^DDB2^ also ubiquitinates DDB2 and XPC. While ubiquitination of DDB2 decreases its DNA binding ability and triggers its destabilization, XPC remains protected owing to RAD23 [6,7,62,63]. A recent report also suggests that DDB2 along with poly(ADP-ribose) polymerase 1 (PARP1) recruits SWI/SNF chromatin remodelling enzyme ALC1 to promote the NER reaction [64].

TC-NER is involved in repairing lesions in transcriptionally active genes. In this process, stalled RNA polymerase II (RNAPII) recruits Cockayne syndrome B (CSB), an SWI/SNF family protein. CSB, in turn, associates with other NER factors, including CSA and p300, which then translocate into the nucleus and colocalize with RNAPII [60]. Similar to DDB2, CSA is directly associated with DDB1–CUL4A complex. So far, CSB is the only known substrate for CSA. CSA and CSB then recruit HMGN1, TFIIS, XAB2 and UVSSA. UVSSA forms a complex with deubiquitinating enzyme USP7 which delays the CSA-dependent degradation of CSB. The lesion is then removed via core NER reaction(s).

Earlier, it was established that CUL4A regulates the abundance of Chk1 in normal cycling cells; however, the identity of the substrate receptor was elusive [11,65]. Recently, it was shown that under replicative stress, CUL4A recruits Cdt2 to target activated Chk1 for proteolysis in a PCNA-independent mechanism [66]. This explains how overexpression of Cdt2 can confer growth advantage in cancers. Recent data also indicate that CRL4A^CDT2^ might also play an important role in post-replication repair by binding to RAD18 and promoting smooth replication via translesion synthesis at regions of spontaneous DNA damage [67]. All these studies imply that CUL4A can be considered as one of the master regulators that control multiple aspects of genomic stability.

### 5.3. Haematopoiesis

CUL4A, which is expressed throughout haematopoietic development, is involved in degradation of multiple HOX proteins such as HOXA9, HOXA1, HOXA2, HOXA11, HOXB4, HOXB7, HOXB8 and HOXB13 [68,69]. HOX genes belong to a family of homeodomain containing transcription factors that play pivotal roles in embryonic development and haematopoiesis [70]. Expression of these genes in haematopoietic stem cells (HSCs) and their progenitors varies in lineage and differentiation stage-specific manner. *Hoxa* and *Hoxb* expression are restricted to HSCs and their precursors, wherein they promote their expansion, and their expression declines upon lineage commitment [71,72]. In bone-marrow-derived diploid 32Dc13 myeloid progenitor cells induced with granulocyte colony-stimulating factor (G-CSF), CUL4A was found to promote granulopoiesis by targeting HOXA9, whereas low levels of CUL4A resulted in HOXA9 accumulation and reduced granulocytic differentiation [69]. Similar results were obtained for HOXB4 [68]. These results indicate that CUL4A might be involved in promoting maturation and differentiation of HSCs. However, the effect of degradation of other HOX proteins by CUL4A on HSCs proliferation and differentiation awaits further investigation.

By contrast, overexpression of CUL4A in the human myelomonoblastic cell line PLB-985, induced with dimethylformamide or phorbol-myristate acetate, was found to attenuate their granulopoietic or monocytopoietic differentiation, respectively [73]. In addition, erythroid cells derived from haploin-sufficient *Cul4A^+/−^* mice showed reduced proliferation and elevated levels of cell cycle regulator p27^Kip1^ [74]. In addition, while ectopic expression of CUL4A in G1E-ER4 proerythroblast cells enhanced their proliferation, it interfered with their maturation and cell cycle exit [74]. In another study, *Cul4A^+/−^* HSCs were found to show defects in engraftment and self-renewal potential [75]. The discrepancy in results might be due to use of different cellular systems in the studies and different pathways being induced. It is also possible that Cul4A might target different regulators in respective cellular systems. Because most of these studies involved use of haplo-insufficient *Cul4A^+/−^* mice, replication of same in *Cul4A^−/−^* mice would conclusively establish the functions. Overall, these findings suggest that a delicate balance of Cul4A is required for normal proliferation, maturation and maintenance of self-renewal capacity of haematopoietic cells. It is also tempting to speculate a potential role of CUL4A in maintenance of cellular stemness.

### 5.4. Spermatogenesis

Initial attempts to create *Cul4A* knockout mice found it to be embryonically lethal [76]. The authors deleted exon 1 of the *Cul4A* gene along with an approximately 1.1 kb upstream sequence. The *Cul4A*^Δ1/Δ1^ embryos, though able to hatch and implant, failed to survive beyond 7.5 dpc. However, it was later discovered that Liu *et al*. [16] had inadvertently deleted the promoter and transcription initiation site of *Psid2* gene present upstream on the complementary strand adjacent to *Cul4A* exon 1. *Psid2* gene codes for a PCI domain-containing protein that is found in the essential subunits of CSN, translation initiation factor 3 and 26S proteasome [77]. In 2009, Liu *et al*. [16] conditionally inactivated *Cul4A* in mice having floxed *Cul4A* exons 17–19, which encodes for ROC/RBX binding site. Conditional deletion of this region in mutant mice exhibited no obvious developmental defects. Another mutant *Cul4A* mouse was developed independently having deletion of exons *Cul4A* 4–8, which encode a portion of the DDB1 binding site, and was surprisingly found to be infertile [26]. Although female *Cul4A*^Δ4–8/Δ4–8^ mice were able to bear and deliver live pups, albeit with low fertility, male *Cul4A*^Δ4–8/Δ4–8^ mice were found to have extremely low sperm counts and defective spermatocytes with compromised motility. Moreover, testes of *Cul4A*^Δ4–8/Δ4–8^ mice exhibited high levels of apoptosis and defective homologous recombination in spermatocytes. It was suggested that this gender-specific discrepancy in effect of *Cul4A* knockout might be due to the low/no compensatory effect of Cul4B, an X-linked gene, in males due to meiotic sex chromosome inactivation. The authors also reported deficiency in DNA double-stranded break (DSB) repair [26]. Later, *Cul4A*^Δ17–19/Δ17–19^ mice were used to generate germ-line-specific deletion of *Cul4A* and similar results were observed, except there were no significant defects in DSB repair [27]. Taken together, these studies identify a novel indispensable role of *Cul4A* in spermatogenesis.

## 6. Regulation of CUL4A

Although CUL4A complex itself is involved in regulation of a myriad of cellular processes, its own activity is tightly regulated by assembly and disassembly cycles mediated by various factors, such as NEDD8 (neural precursor cell-expressed developmentally downregulated protein 8, CSN and CAND1 (cullin associated NEDD8-dissociated 1. Dimerization of CRL4A is also believed to play an important role in its regulation.

NEDD8 is the ubiquitin-like protein whose conjugation with cullins, referred to as neddylation, stimulates their ubiquitin ligase activity. Neddylation of cullins has been shown to promote conformational change in E3 complex structure such that E2-Ub gets positioned adjacent to the substrate for effective ubiquitin transfer [3,78,79]. Deneddylation of cullins is mediated by CSN, an evolutionarily conserved eight subunit complex containing Nedd8 isopeptidase activity [80,81]. CSN inhibits autoubiquitination of DCAF in non-enzymatic fashion and this inhibition is relieved upon DCAF binding to substrate, which subsequently causes CRL activation [33]. When deneddylated, cullins are sequestered by a 120 kDa protein called CAND1 [82,83]. Although *in vitro* CAND1 binds to all cullins, *in vivo* it has been found to interact with CUL 1, 2, 3 and 4A in human HeLa cells [83] and CUL 1, 4A and 5 in HEK293T cells [84]. Intriguingly, CAND1 was found to inhibit CRL ubiquitination activity *in vitro*; however, *in vivo* it promoted CRL activity. This paradox was finally resolved recently when it was shown that CAND1 functions in substrate receptor exchange cycles on CUL1, which can also be expected to be similar for other cullins. According to this model, in saturating substrate concentration, the neddylated form of cullin possesses high affinity for its adaptor–SR complex and very low affinity for CSN. In such conditions, substrate meets its fate depending upon its ubiquitination pattern. However, once substrate is depleted, CSN affinity for CRL complex increases, and it is able to dislodge Nedd8. In this metastable transition state, depending upon the cellular conditions, cullin–adaptor–SR complex can (i) bind to new substrate and undergo neddylation to return to the ‘active ubiquitination state’ or (ii) enter an ‘exchange state’ to form a transient complex with CAND1 which leads to dissociation of adaptor–SR complex. In the latter case, CAND1–cullin–ROC1 complex then binds to new adaptor–SR complex to form an unstable ternary intermediate state having stearic interference between CAND1 and cullin bound adaptor–SR complex. This ‘exchange regimen’ can either yield new CRL complex or the intermediate state decays back to CAND1–cullin–Rbx complex [19–21]. Thus, CAND1 and CSN can influence the function of CRLs by altering the neddylation status of cullins, thereby remodelling the E3 complexes and regulating the association and dissociation of substrate adaptors.

There may be additional proteins that interact with cullin complexes to regulate their function, e.g. DDA1 (DDB1- and DET1-associated factor) interacts with CUL4A–DDB1 complex, but the importance of this interaction still needs to be determined [31,85]. It is also speculated that dimerization of CRL4A/4B through Nedd8 or substrate receptor may also play an important role in regulating its activity [67].

**Figure 4.**
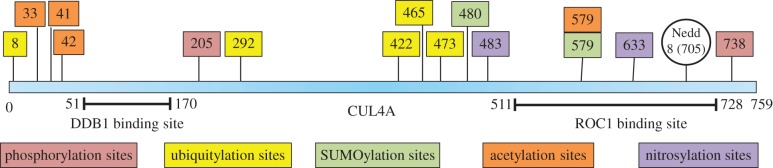
Potential PTM sites on CUL4A protein. These potential PTM sites were found using various tools. Validity of these sites warrants further investigation.

Because PTMs are known to play a central role in imparting dynamic functions to proteins and create diversity in signalling, we investigated the possible modifications of CUL4A. So far, neddylation is the only modification reported, thus we performed several bioinformatics analysis of CUL4A primary sequence and mapped various potential sites for PTM, which were found to be conserved across species. These potential PTM sites were found using the following tools: for phosphorylation, disorder-enhanced phosphorylation sites predictor (DISPHOS) [86], NetPhos 2.0 [87] and Phosida [88]; for ubiquitination, UbPred [89], BDM-PUB (http://bdmpub.biocuckoo.org/) and CKSAAP [90]; for SUMOylation, SUMOplot (http://www.abgent.com/sumoplot) and SUMOsp 2.0 [91]; for acetylation, ASEB [92] and Phosida [88]; and for nitrosylation, GPS-SNO 1.0 [93] and iSNO-PseAAC [94]. The sites which consistently turned up in all the tools were taken into consideration, and a schematic model was drawn (figure 4). We hypothesize that some of these potential PTM sites may be involved in regulation of CUL4A function possibly by altering its localization or stability or interaction with other proteins. Because PTM at one site can promote or inhibit PTM at an other site on a protein, it is probable that cross-talk between these modifications may also be involved in regulating CUL4A function. For example, modifications such as ubiquitination, SUMOylation, methylation and acetylation may compete for certain lysine residues; SUMOylation at one site, say position 480, may make other potential ubiquitination sites more prone to get ubiquitinated, thereby altering the stability or function of CUL4A. As a result, function of CUL4A may depend on its net multisite PTM profile. Thus, investigations towards verification of these PTMs, demonstration of temporal and spatial dynamics of these modifications *in vivo* and assignment of biological functions to these PTMs may shed light on the molecular mechanism of action of CUL4A and its interacting partners.

## 7. Deregulation of *CUL4A* leads to tumourigenesis

Owing to its critical role in cell cycle regulation and genomic stability, any deregulation in *CUL4A* copy number or expression is expected to result in a profound effect on cells. Human chromosomal region 13q34 appears to be one of the hot spots in cancers that undergoes amplification ([8] and references therein). These high level gains may help cancer cells to upregulate genes that may drive tumourigenesis. *CUL4A* has been found to be amplified in squamous cell carcinoma [95], adrenocortical carcinomas [96] and childhood medulloblastoma [97]. Its amplification and overexpression was also found in hepatocellular carcinomas [8], primary malignant pleural mesotheliomas [98], primary human breast cancers [2] and prostate cancers [99]. A recent study also observed overexpression of *CUL4* in epithelial ovarian tumours especially in the invasive carcinoma specimens [100]. High *CUL4A* expression correlates with accelerated neoplastic transformation along with significantly shorter overall and disease-free survival in node-negative breast cancers and ovarian tumours [14,100]. Furthermore, conditional overexpression of *CUL4A* in lungs of transgenic mice leads to development of pulmonary hyperplasia [101], while *Cul4A*^Δ17–19/Δ17–19^ mice were found to be hyper-resistant to UV-B-induced skin carcinogenesis compared with wild-type and heterozygotes [16]. Recent evidence also highlights CUL4A's essential role in ubiquitination of several well-defined tumour suppressor genes. In unstressed cells, CRL4^CDT2^ associated with MDM2 and p53, in a PCNA-dependent manner, to bring about the polyubiquitination and degradation of the latter. However, upon UV-irradiation CRL4^CDT2^ affinity for p53 attenuates, leading to its stabilization [10,102]. CUL4A also brings about inactivation of transcriptional function of p73, a structural and functional homologue of p53 [103]. This repression was found to correlate with overexpression of CUL4A in human breast carcinoma [103]. Additionally, CUL4A targets p150/Sal2 for degradation when cells transit from quiescence to mitotic state [104]. Furthermore, RAS association domain family 1, isoform 1 (RASSF1A), a mitotic regulator and tumour suppressor, undergoes CUL4A–DDB1 complex-mediated proteolysis during the M phase of the cell cycle [105]. In addition, p21, the master effector of multiple tumour suppressor pathways, has been shown to accumulate in *Cul4A* deleted MEFs upon UV irradiation leading to prolonged G1/S arrest [16]. CRL4^β-TrCP^ and CRL4^Fbw5^ also target mTOR signalling inhibitors REDD1 and Tsc2, respectively [106,107]. Taken together, these studies highlight the importance of CUL4A in promoting tumourigenesis (see also table 1). However, there are also reports wherein CUL4A has also been shown to target proto-oncogenic targets such as N- and C-Myc and c-Jun by recruiting TRCP4AP/TRUSS and COP1, respectively [109,110].

**Table 1.** CUL4A interacting partners with proven role in tumourigenesis.

interacting partner	function	reference
Chk1	Ser/Thr kinase involved in cell cycle arrest following DNA damage	[11,65,66]
p27^KIP1^	cyclin-dependent kinase inhibitor involved in cell cycle arrest	[39–41]
HOXA9	transcription factor involved in morphogenesis and differentiation	[69]
ETV1	transcription factor belonging to ETS (E twenty-six) family	[108]
p53	tumour suppressor involved in cellular response to DNA damage	[10,102]
c-Jun	component of transcription factor AP-1	[109]
N-Myc, C-Myc	transcription factor involved in cell proliferation and apoptosis	[110]
RASSF1A	potential tumour suppressor	[105]
p150 (ABL1)	proto-oncogenic tyrosine kinase	[104]
XPC	nucleotide excision repair	[63]

## 8. Exploitation of CRL4A by viral proteins

**Figure 5.**
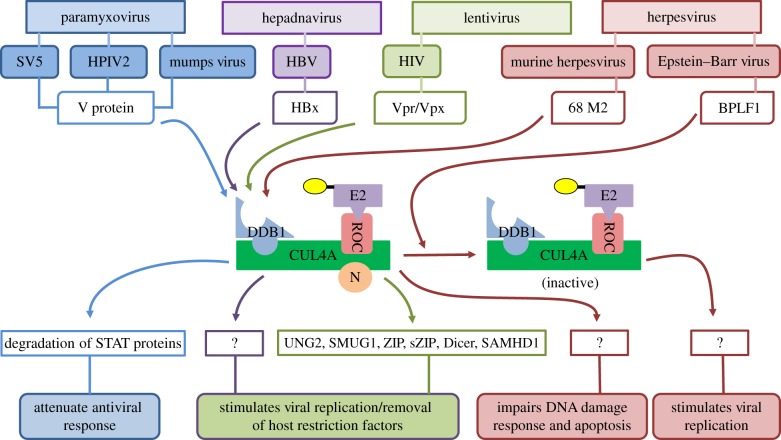
Hijacking of cellular CUL4A machinery by viruses. Members of various viral families target the CUL4A–DDB1 complex to facilitate their own replication and dissemination. N, Nedd8.

Manipulation of host CRLs is one of the common strategies used by pathogenic viruses to override host factors that may prevent or impede their infection [111–113]. Proteins encoded by members of paramyxovirus, herpesvirus, lentivirus and hepadnavirus families are known to target CUL4A machinery (figure 5). Paramyxovirus V protein from Simian virus 5 (SV5) and human parainfluenza virus type 2 (HPIV2) interact with host CRL4A, forming V-dependent degradation complex (VDC), to recruit STAT1–STAT2 heterodimer for degradation [114]. Similarly, mumps virus V protein uses VDC to additionally degrade STAT3 protein [115]. Later, crystal structure of DDB1 in complex with SV5-V protein showed that viral V protein inserts its N-terminal α1-helix into BPA–BPC double propeller. This configuration allows it to recruit STAT1–STAT2 heterodimer for ubiquitination and subsequent degradation, thereby attenuating the interferon pathway of antiviral response [28]. Hepatitis B virus (HBV), which is one of the primary cause of liver diseases such as cirrhosis and hepatocellular carcinoma, uses X protein (HBx) to hijack DDB1–Cul4A complex [116,117]. Structural analysis of CUL4A–DDB1–HBx revealed that HBx adopts a helical structure similar to SV5 V protein, which it slips into BPA–BPC along with its H-box motif which docks on the top surface of the DDB1–BPC domain [118]. Although the cellular targets of CUL4A–DDB1–HBx are yet to be identified, it has been shown that this interaction promotes viral replication and leads to stabilization of proto-oncogene pituitary tumour-transforming gene 1 (PTTG1), which is overexpressed in hepatocellular carcinoma [119–121]. Additionally, despite having any homology with SV5V protein or HBx protein, M2 protein of murine γ-herpesvirus 68 (γHV68) was found to interact with DDB1 resulting in inhibition of DNA-damaged-induced apoptosis, which may help in viral latency [122]. In addition, Epstein–Barr virus (EBV) large tegument protein BPLF1 has been found to remove NEDD8 from Cul4A by using its DUB, thereby stabilizing the CDT1 and pushing the host cell towards S phase [123]. Finally, Vpr and its paralogue Vpx, small encapsidated accessory proteins of HIV, with the former being shared by HIV-1 and HIV-2 and the latter being exclusive to HIV-2/SIV, associate with VprBP/DCAF1, a substrate receptor of CUL4 complex [15,124]. Uracil DNA glycosylases UNG2 and SMUG1 and transcriptional regulators ZIP and sZIP and Dicer are the only known substrates of HIV-1 Vpr–CRL4^VprBP^-mediated degradation [125–128]. However, the significance of targeting these substrates and bringing about G_2_ arrest for viral propagation still remains to be ascertained [15]. On the other hand, HIV-2 Vpx recruits CRL4^VprBP^ to target SAMHD1 (SAM domain and HD domain-containing protein 1) to facilitate HIV-2 invasion in macrophages and dendritic cells [124,129–131]. SAMHD1 is a deoxynucleoside triphosphate triphosphohydrolase that depletes the dNTP pool in non-dividing cells, thereby impeding viral replication [132]. Thus, Cul4A–DDB1 complex seems to be an attractive target for viruses, and elucidation of mechanisms of CUL4A hijacking and their significance for the survival of viruses can help in developing better therapeutic strategies against HIV and other viral infections.

## 9. Promising prospects of *CUL4A* in diagnosis, prognosis and treatment of cancer

Recent studies (discussed above) clearly identify CUL4A as a potential candidate gene for cancer progression. Thus, CUL4A can be a potential target for developing anti-cancer therapeutics because of the following reasons: first, it has been found to be overexpressed in multiple cancers and implicated to play a role in carcinogenesis [2,8,95–100]; second, its overexpression correlates with poor prognosis of patient survival [14,100]; and third, knockdown of its expression leads to inhibition of cancer cell growth and apoptosis and, conversely, its overexpression leads to formation of pulmonary hyperplasias [16,101]. This indicates that CUL4A can be a promising anti-cancer target.

Evidence also suggests that CUL4A levels can be used as a biomarker for predicting whether a patient will respond to a particular therapeutic. It was shown that high CUL4A expression levels confer prostate and breast cancer cell sensitivity to thalidomide and trabectedin, respectively [99,133]. Thus, screening for CUL4A levels in cancer patients may help in achieving better drug response with minimal unwanted side effects.

A dysfunctional UPS has been associated with multiple cancers, wherein it degrades various cell cycle inhibitors and apoptotic proteins, thereby helping the tumour cells to evade apoptosis and undergo uncontrolled division. Thus, UPS represent an attractive potential target for anti-cancer therapeutics. This led to the approval of the first and only proteasome inhibitor, bortezomib (also known as Velcade or PS-341) by the US Food and Drug Administration, further driving the interest in the development of anti-cancer drugs targeting the UPS [134,135]. Bortezomib has been approved for the treatment of multiple myeloma and mantle cell lymphoma. However, its clinical use is hampered by substantial toxicity, other side effects including inhibition of a multitude of proteins involved in various processes [136–138]. Hence, it is predicted that such general proteasome inhibitors may have a very narrow therapeutic window. Therefore, there is a pressing need for developing inhibitors which specifically target a particular aspect of the UPS pathway, thereby moderating the deregulated pathway of cancer cells. Because specificity of the UPS pathway is dictated by E3 ligases, they represent an appealing target for developing anti-cancer therapies.

MLN4924, a small molecule inhibitor of NEDD8 activating enzyme (NAE) has entered phase I clinical trials for haematological and solid tumour malignancies [139]. MLN4924 specifically prevents NEDD8 modification of cullins, thereby selectively attenuating their activity. It has already shown promising growth inhibitory properties in cancer cell lines derived from colon, lung, myeloma and lymphoma and in xenograft models. Although compared with bortezomib MLN4924 appears to be a better candidate drug as it targets particular superfamily of E3 ligases, it would be judicious to wait for the results of phase I clinical trials to see whether it exhibits any serious side effects.

More selective E3 ligase targeting molecules are Nutlins, which are considered *bona fide* inhibitors of p53 and MDM2 interaction [140]. These molecules are *cis*-imidazoline analogues that compete for p53 binding site on MDM2, thereby leading to p53 stabilization, cell cycle arrest and apoptosis [140]. As a result, this class of molecules has shown promising anti-cancer efficacy in cancer cell line xenograft assays. Examples include Nutlin-3 and its pharmacologically optimized form, RG7112, which are currently undergoing phase I clinical trials for the treatment of retinoblastoma and liposarcomas, and haematological malignancies, respectively [141,142]. Owing to their selective nature, Nutlin-3 and RG7112 are expected to have less deleterious effects on healthy tissues, although the real scenario will only be clear once the results of the clinical trials are published.

## 10. Perspective

As a result of intense research effort, today we know that CUL4A ubiquitin ligase plays a key role in a wide range of cellular processes, including the cell cycle, chromatin remodelling, DNA damage response, DNA replication, spermatogenesis and haematopoiesis. On the pathology front, CUL4A is attacked by several viral proteins, and its overexpression is a common feature of many human cancers. Considering the significance of CUL4A complexes in assorted cellular functions and how perturbation in its expression or function leads to pathologies, it represents an attractive target for drug discovery efforts. However, discovery of highly specific inhibitors remains a challenging task. With increased understanding of CUL4A's physiological partners, biological functions, molecular mechanism of action and structure–function relationships, and with the availability of advanced research technologies, more selective CUL4A-directed therapeutics are expected to be discovered. This calls for extensive research in this emerging area involving functional delineation of CUL4A adaptors and substrates and study of deregulated pathways leading to human diseases.

## Figures and Tables

**Figure 6.**
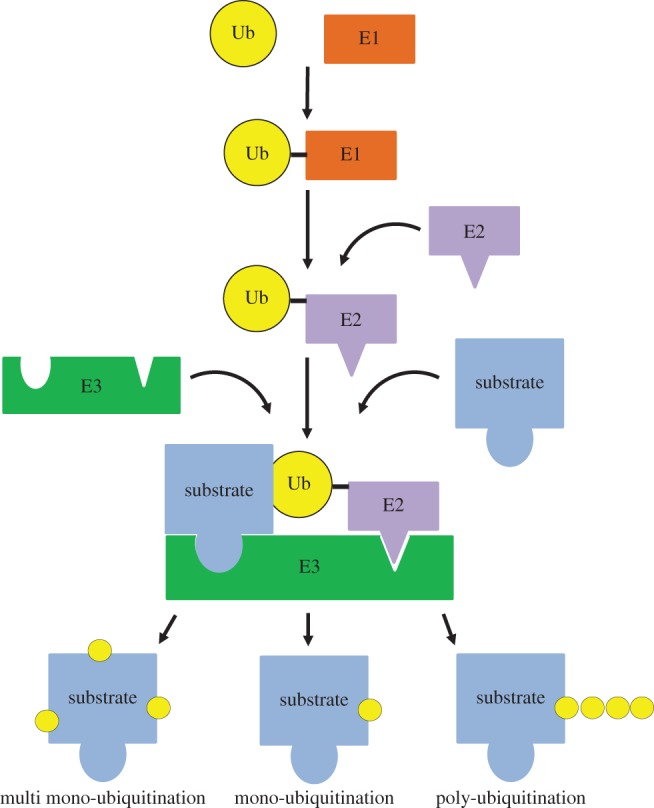
Ubiquitination process. The cascade of E1–E2–E3 enzymes tags a substrate with ubiquitin. The fate of substrate depends upon the type of ubiquitination it undergoes.

## Appendix A. Ubiquitin–proteasome system

The ubiquitin–proteasome system (UPS) is one of the major proteolytic systems involved in vital processes such as protein quality control, stress response, homeostasis and cell survival [143–145]. It functions by covalent tagging of substrate protein with ubiquitin (Ub) via enzymatic cascade involving thioesterification reactions [146–148]. The process involves two E1 enzymes, about 40 E2s and nearly 600 E3s in the case of humans [149–152]. First, ubiquitin activating enzyme, E1, forms a thioester linkage with the C-terminal glycine residue of Ub in an ATP-dependent manner. Then, again via thioester linkage, Ub is transferred to ubiquitin conjugating enzyme, E2. Finally, E2 enzyme binds to ubiquitin ligase enzyme, E3, and the complex mediates isopeptide linkage formation between carboxy terminal glycine residue of Ub and lysine *ɛ*-amino group of the substrate [146,148]. Repetition of this catalytic cycle leads to polyubiquitination of the substrate [153]. Ub can bind to the substrate either through its N-terminal or other internal lysine residues (K6, K11, K27, K29, K33, K48 and K63). While monoubiquitination alters the cellular localization or protein–protein interactions, substrates bound to Ub chains of four or more residues through K11 and K48 of Ub are marked for proteasomal degradation. The role of other atypical linkages such as branched chains, mixed chains or multiple monoubiquitination is still being deduced (figure 6) [154,155].
